# Pilot-testing a Multiplayer HIV and Sexually Transmitted Infection Prevention Video Game Intervention for Black Adolescent Girls: Protocol for a Randomized Controlled Trial

**DOI:** 10.2196/43666

**Published:** 2023-01-23

**Authors:** Veronica Weser, Ijeoma Opara, Mariana Budge, Lindsay Duncan, Claudia-Santi F Fernandes, Sydney Hussett-Richardson, Brandon Sands, Kimberly Hieftje

**Affiliations:** 1 Yale School of Medicine New Haven, CT United States; 2 Yale School of Public Health New Haven, CT United States; 3 Department of Kinesiology and Physical Education McGill University Montreal, QC Canada

**Keywords:** black, adolescent, female, video games, feasibility studies, HIV prevention, randomized controlled trial, sexual health, condom use, self-efficacy, pre-exposure prophylaxis, PrEP

## Abstract

**Background:**

Black adolescent girls aged between 14 and 19 years are more likely than White girls to be diagnosed with a sexually transmitted infection (STI). As STI diagnosis is associated with an increase in the risk for HIV acquisition, an early intervention specifically tailored to Black adolescent girls is warranted. A web-based video game intervention has the potential to reach this demographic. Because studies of social and behavioral determinants of disease demonstrate the protective role of peer group structures on individual outcomes, a multiplayer game can facilitate opportunities to exchange and evaluate information, learn social norms, develop behavioral skills, and allow peers to influence attitudes and behavior. No prior research has examined the feasibility of a web-based multiplayer game intervention for this population.

**Objective:**

This study describes the protocol for a randomized controlled trial (RCT) pilot-testing the feasibility, acceptability, and limited efficacy of a multiplayer game–based intervention for increasing HIV and STI testing and condom use in Black adolescent girls.

**Methods:**

We enrolled 79 Black adolescent girls aged 14 to 19 years residing in the United States into a 2-arm parallel RCT. The intervention is a theory-based, community-informed, multiplayer game that can be played with peers on the web using videoconferencing software. The goal of the game is to empower Black adolescent girls to make healthy decisions regarding dating, relationships, and sex, thus reducing HIV and STI infection. Control condition participants received a list of resources after playing a time and attention control game. All study procedures were conducted via the internet. We conveniently sampled Black adolescent girls using web-based advertisements. Study assessments occurred at enrollment, 1 week, 1 month, and 4 months after enrollment. The primary outcome of this study is increased HIV and STI testing by Black adolescent girls. Secondary outcomes include increased condom use, self-efficacy to use condoms, positive attitudes toward condom use, intentions, harm perceptions, HIV and STI and pre-exposure prophylaxis knowledge, positive sexual norms, sexual communication with partners, and reduced incidence of sexual risk behaviors associated with HIV and STI transmission. Secondary outcomes also included assessment of intervention feasibility and acceptability.

**Results:**

From February to April 2022, a total of 79 Black adolescent girls were enrolled, with 40 (51%) having been randomized into the intervention condition and 39 (49%) into the control condition. At baseline, participant ages ranged from 14 to 19 (mean 16.4, SD 1.23) years.

**Conclusions:**

Web-accessible game interventions overcome common impediments of face-to-face interventions presenting a unique opportunity to reach Black adolescent girls and improve their sexual health and self-efficacy. Trial data will provide information about the limited efficacy of the intervention and inform future web-based studies and a larger RCT aimed at improving the sexual health of Black adolescent girls.

**Trial Registration:**

ClinicalTrials.gov NCT04108988; https://clinicaltrials.gov/ct2/show/NCT04108988

**International Registered Report Identifier (IRRID):**

DERR1-10.2196/43666

## Introduction

### Background

Despite advances in HIV prevention efforts, Black adolescents continue to be disproportionately affected by HIV in the United States, comprising 54% of all new HIV diagnoses in 2016 [1]. In the United States, Black adolescent girls aged between 14 and 19 years have higher rates of sexually transmitted infections (STIs) than other ethnic minority girls and are more than twice as likely to be diagnosed with an STI than their White peers [2-5]. Racist, sexist, and classist views significantly disadvantage Black adolescent girls’ achievement of positive health outcomes relative to White girls [6]. Indeed, mounting evidence implicates the role of gendered-racist stereotypes on increases in Black girls’ sexual risk behaviors [7,8], indicating a need for the development of HIV and STI prevention strategies specifically tailored for Black adolescent girls.

A recent systematic review noted that many existing sexual health promotion interventions for Black youth are not specifically tailored for Black adolescent girls or made to account for their unique experiences because of the intersection of race and gender [9]. Because increased knowledge about HIV and HIV prevention strategies is associated with protective sexual behaviors such as condom use and HIV testing among adolescents of color [10,11], there exists a critical need for the development of more interventions specifically designed for Black adolescent girls that increase the knowledge about HIV and STI prevention and emphasize resisting and challenging gendered-racist stereotypes [12]. Although the recent systematic review identified 15 effective HIV and STI prevention programs that have been used with Black adolescent girls, only 4 were created specifically for this population [9]. In addition, challenges regarding implementation and fidelity of interventions have been a concern. Studies have cited several barriers to the implementation of HIV and STI prevention interventions, including access to adequately trained providers, staff turnover, resource constraints of agencies, and fidelity of the interventions [13-15].

One possible method for intervention delivery is through the creation of a self-contained video game, which can provide consistent fidelity, and places little demand on program resources [16,17]. Game interventions have demonstrated efficacy in increasing knowledge and affecting behaviors related to health promotion and disease management across domains [18]. Successful game interventions have focused on health topics ranging from asthma management, healthy diets, medication adherence, and even sexual health promotion through risk behavior reduction [19-22]. Because 83% of adolescent girls play video games on computers, web, phone, or console [23], a game-based HIV and STI prevention intervention specifically tailored for Black adolescent girls will likely be an acceptable and enjoyable means of delivering the material.

Among adolescent girls, social or multiplayer games are among the most popular and frequently played video games [24]. Because interactions within peer groups offer opportunities for individuals to exchange and evaluate information, learn social norms, develop behavioral skills, and influence each other’s attitudes and behavior [25], an HIV and STI prevention initiative that targets peer groups by leveraging adolescent girls’ preference for multiplayer gaming experiences has the potential to have benefits above and beyond an intervention designed for a single individual. Indeed, research suggests that peers often serve as a valuable source of information about HIV and as role models for prevention strategies such as condom use [26]. For instance, a community-based pilot study demonstrated a 72% increase in the number of individuals getting tested for HIV and STI when peers recruited others through their social network [27]. Similarly, a recent study of at-risk Black and Hispanic youth aged 13 to 24 years found that a female friendship network method of engagement was successful with increasing HIV testing rates by 90% [28].

Although Boyd et al [29] found that Black and Latinx adolescents who learned about HIV from their friends were *less* likely to get tested for HIV, the researchers note that youth surveyed in their study likely perceived the HIV-related knowledge gleaned from friends as unreliable. Therefore, to take advantage of the benefits that peer group structures can have on individual determinants of health, it is important that a multiplayer intervention create a shared experience where the information provided is known by all to be both factual and relevant to the players’ lived experiences. Adopting this strategy in the creation of an HIV and STI prevention multiplayer video game specifically tailored for Black adolescent girls allows for conversations between players to occur in real time during gameplay, which deepens engagement with the material and provides girls the opportunity to positively influence attitudes, beliefs, and perceptions around serious topics. Moreover, the game environment encourages a safe and playful space where peer groups can normalize discussions about sex and safe sex practices. Because most research on Black girls’ sexuality and sexual health is focused on the reduction of risky behaviors rather than fostering safe and healthy sex and sex positivity [12,30], a game-based sex positive intervention represents a novel strategy. Such an intervention has the potential to impact the sexual self-efficacy of Black adolescent girls, high levels of which have been associated with HIV-risk mitigation strategies such as consistent condom use by adult Black women [31].

In light of the COVID-19 pandemic, efforts to recruit groups of Black adolescent girls to gather in person for multiple gameplay sessions were stymied. Thus, rather than continue to delay data collection, the research team used their extensive experience working with youth and decided to pivot to web-based data collection by following a model previously found successful in the collection of focus group data that served as the foundation for the development of this study’s video game intervention [32,33]. This decision was made with knowledge from conducting numerous randomized controlled trials (RCTs) with schools and informed by processes that we developed to accommodate remote data collection.

With the ubiquity of the videoconferencing software Zoom (Zoom Video Communications, Inc) and recent reports that “gamified videoconferences” facilitated both connection and learning during periods of social isolation [34], the research team embraced the opportunity to recruit a sample of Black adolescent girls from across the United States, rather than wait for the resumption of in-person learning at local school partner sites. Although web-accessible game interventions overcome many common barriers of face-to-face interventions and offer a unique opportunity to reach Black adolescent girls across the United States, no previous studies have examined the use of multiplayer game interventions delivered remotely to this population. Therefore, pilot-testing the limited efficacy of this specifically tailored multiplayer game intervention is necessary to understand the acceptability of the gaming experience such that future expansions and adaptations can be created. Moreover, results of this pilot limited efficacy test can be used to estimate variability and precision to power a future larger RCT.

### Study Aims

#### Primary Aim

Our primary aim of this study is to evaluate the feasibility and acceptability of a multiplayer game–based intervention designed to increase HIV and STI testing and condom use among adolescent Black girls.

Feasibility of this intervention is assessed by collecting data on the number of intervention sessions completed by participants and retention rates. We hypothesize that the intervention will have high completion and retention rates across survey time points (>80%).

Acceptability of the intervention condition is assessed via self-reported survey at the postgameplay time point using the game experience and satisfaction questionnaire, which is a 15-item scale with 4-point Likert-type responses ranging from 1 (strongly disagree) to 4 (strongly agree) [22,35-37]. Example questions include “I enjoyed playing the game” and “I would make decisions in life like I made them in the game.” We hypothesize that in response to the question as to whether a participant would be interested in playing the game again and whether they would recommend the game to their friends, ≥75% of participants will “agree” or “strongly agree” that they would like to play the game again and ≥75% will “definitely” or “very probably” recommend the game to their friends.

#### Secondary Aim

Our secondary aim of this study is to determine the preliminary impact of the intervention on knowledge, intentions, beliefs and attitudes, self-efficacy, social norms, and behavioral skills related to HIV and STI testing, condom use, and sexual risk behavior reduction. We hypothesize that compared with control participants, participants in the intervention group will have greater improvements in all proximal outcomes.

#### Exploratory Aim

This study includes an exploratory aim designed to evaluate participants’ real time responses to the game through the content analysis of audio recordings of gameplay sessions. We will investigate participant perceptions of research procedures and game content to analyze suggestions for the improvement of both. We will also explore whether connection to one’s ethnic identity may be a protective factor against sexual risk behavior [38]. Because the exploratory aim concerns implementation procedures and the adaptation and expansion of the game, there are no a priori hypotheses.

## Methods

### Ethics Approval

All the procedures were approved by the Yale University Institutional Review Board (ID #2000026487). In addition, a Data Safety and Monitoring Board consisting of 2 experts in clinical trials in adolescents and in statistical analysis of clinical trials was formed to oversee the safety and data integrity of this study. The Data Safety and Monitoring Board also periodically reviewed the progress of the study. The following personally identifying and medical information were collected: email ID, phone number, self-reported HIV and STI testing status, test results, and sexual activity status. All data collected from the assessments were entered by participants directly into the secure web-based system, Qualtrics (Qualtrics International Inc), which is Yale’s enterprise-wide data management system. This ensured participant confidence that their data would remain confidential. Study participation incentives were sent via email as Amazon e-gift cards. Written consent was obtained via digital signature from all the participants before their participation, as detailed in the section Study Population and Study Flow.

### Study Design

This study is a pilot of a 2-arm RCT designed to evaluate the feasibility and acceptability of a multiplayer game–based intervention designed to increase HIV and STI testing and condom use among adolescent Black girls. The study is led by a team with expertise in conducting research with youth, as well as extensive experience with the design and evaluation of game-based health promotion interventions, including interventions specifically designed for HIV and STI prevention [22,37]. Our multiplayer video game intervention prototype and research protocol were developed through extensive collaboration with partners including a specialist in strength-based approaches to understanding HIV and STI disparities among girls and women of color, a biostatistician, and a professional game development company, PreviewLabs, Inc.

### Study Population and Study Flow

**Figure 1:**
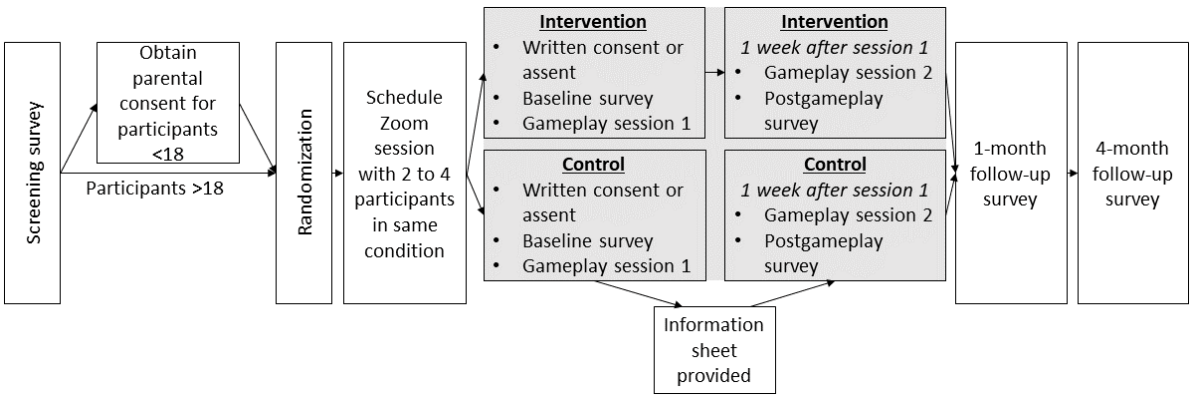
Two-arm randomized controlled trial design and data collection schedule.

All study procedures including screening, consenting, and surveying were conducted through Qualtrics, a secure Health Insurance Portability and Accountability Act–compliant website for managing web-based surveys and databases. Study participant flow is depicted in Figure 1.

First, to determine eligibility, respondents completed a brief web-based self-reported screening questionnaire, the link to which was displayed on the social media advertisements. Respondents were eligible if they met the following eligibility criteria: they must (1) be aged between 14 and 18 years; (2) identify as Black; (3) identify as female; and (4) identify as straight (ie, heterosexual), bisexual, or pansexual. Once ≥3 prospective participants signed up for a single study session, the project manager or research assistant emailed those 3 prospective participants the Zoom link for the study session and a description of the procedure, which included recommendations for equipment (a PC for Zoom and cell phone for gameplay). Respondents under 18 years of age were also required to provide an email address for a parent or guardian.

All the eligible respondents were assigned a participant number and were randomly assigned to either the experimental or control condition (full descriptions of conditions are provided in later sections). Eligible respondents then received an email from the project manager or research assistant with details about the study and exclusion and inclusion criteria, and for respondents under the age of 18 years, the parental information sheet and a link to the informed consent document was emailed to the parent or guardian using the address provided in the screening survey. Prospective participants who were 18 years old and respondents whose parent or guardian digitally signed the informed consent document were then invited to sign up for a study session.

Separate study sessions for experimental and control participants were determined each week by the project manager and research assistant. Prospective participants were instructed to indicate their availability for as many study sessions as possible. Respondents provided their email address and had the option to provide a cell phone number if they preferred to receive text messages for study communication. If additional prospective participants later indicated availability for a study session for which links had already been distributed, those participants were similarly provided with the Zoom link and study procedure description. Invitations to study sessions were capped at 8 prospective participants per session.

Adolescent assent for participants under 18 years and informed consent for participants aged 18 years were obtained at the start of each Zoom study session. After explaining the risks and requirements of the study in general terms, the researcher provided each participant a link to the full consent or assent document via Qualtrics. Participants were given ample time to fully read through the document and asked to provide their digital signature if they agreed to participate. The researcher was available to answer all the questions regarding the consent and assent document. Following consent procedures, the baseline survey was administered (full description of the baseline survey is provided in a later section). Once all participants had completed the baseline survey, the researcher launched the video game according to condition (intervention game vs control game; both described in detail in a later section). Throughout gameplay, participants were encouraged to use Zoom’s chat feature to discuss the game.

Upon conclusion of the first gameplay session, participants were emailed an Amazon gift card worth US $30 and a second gameplay session at the same time in the following week was scheduled. Participants who were unable to attend a second gameplay session at the same time in the following week were scheduled for a different second gameplay session with another group via email. For all the participants, the second gameplay session began with playing either the control or the experimental game, followed by the completion of the postgameplay survey (full description of the postgameplay survey is provided in a later section). Once all the participants submitted their postgameplay survey, a second Amazon gift card worth US $30 was emailed to all participants.

The first follow-up survey was emailed to all the participants 1 month after their second gameplay session. The second follow-up survey was emailed to all the participants 3 months after the completion of their first follow-up survey. For each follow-up survey completed, participants received a US $30 Amazon gift card via email. Participants who did not complete either follow-up survey within 5 days of receiving the survey were reminded to complete the survey via email. An additional email reminder was sent 1 week after the first reminder. Participants who asked to be contacted via text message additionally received survey links and reminders through text message.

### Recruitment

Participants were conveniently sampled and recruited throughout the United States using advertisements on Facebook and Instagram. Instagram was our primary recruitment site because it is possible to target individuals of a particular age (14-18 years) and gender (female) through the advertising platform. Moreover, Instagram is used by approximately 71% of adolescents, making it an appropriate platform to reach our target demographic [23]. We created an advertisement and recruited participants from Facebook using the same advertisement and targeting criteria. An individual who was interested in participating clicked on the advertisement and was directed to the Qualtrics screening survey.

### Assessments

To evaluate the multiplayer game–based intervention we collected assessment data at 4 time points during the study: baseline, after the gameplay, 1-month follow-up, and 4-month follow-up. The assessments administered at each time point are depicted in Table 1.

**Table 1:** Assessments and time of administration.

Assessment	Baseline	Postgameplay time point	1-month follow-up	4-month follow-up
Participant demographics (ie, gender, age, and sexual orientation)	✓			
Self-reported HIV and STI^a^ testing rate (1 item)	✓	✓	✓	✓
Sexual Risk Behaviors and Sexual communication—self-reported measures of sexual intercourse with or without condoms and frequency of safe sex discussions with partners (27 items)	✓	✓	✓	✓
CUSES^b^ (11-item Likert scale)	✓	✓	✓	✓
HIV and STI KQ^c^ (12 items true or false questions adapted from HIV-KQ and STD-KQ)	✓	✓	✓	✓
PrEP^d^ knowledge and attitudes (11 items)	✓	✓	✓	✓
Microbehaviors: discussing condoms with partners, purchasing condoms, and discussing testing with partners (4 items)	✓	✓	✓	✓
MEIM-R^e^ (6-item Likert scale)	✓			✓
Self-efficacy in sexual risk behaviors—SRBBS^f^ (27-item Likert scale)	✓	✓	✓	✓
Gameplay experience and satisfaction; *intervention participants only* (12-item Likert scale)		✓		
Game experience reflection; *intervention participants only* (4 items)			✓	✓

^a^STI: sexually transmitted infection.

^b^CUSES: Condom Use Self-Efficacy Scale.

^c^KQ: Knowledge Questionnaire.

^d^PrEP: pre-exposure prophylaxis.

^e^MEIM-R: Multigroup Ethnic Identity Measure—Revised.

^f^SRBBS: Sexual Risk Behavior Beliefs and Self-efficacy.

### Materials

#### Control Materials

Participants randomized into the control condition attended live Zoom sessions with up to 4 other control participants and a researcher, who facilitated gameplay of the non–health-related attention and time control condition multiplayer video game. Players in control Zoom sessions played 1 of 2 Jackbox (Jackbox Games, Inc) games (Fibbage or Drawful), which were selected for use as attention and time control games for their similarity in game structure to the intervention game. Participants randomized to the control condition received a short list of links to resources with HIV- and STI-related general information, prevention, testing locations searchable via zip code, and suggestions for talking with partners about HIV and STI testing and prevention strategies such as condom use via email. Finally, after completion of the final 4-month follow-up survey, control participants were offered access to a web page that contained the information and resources provided in the intervention. Participants in the control condition played 2 full games of Jackbox during each gameplay session, resulting in 100 to 120 minutes of total playtime.

#### Intervention Materials

Participants randomized into the intervention condition attended live Zoom sessions with up to 4 other intervention participants and a researcher, who facilitated gameplay of the multiplayer intervention video game, *InvestiDate*. The design and story content of the intervention video game was based on information the research team gathered through focus groups with 27 Black adolescent girls in which participants discussed their experiences with sex, dating, the use of social media to inform partner selection, and participants’ experiences of gendered-racist stereotypes and how these experiences influenced their own sexual decision-making [32,33]. In addition, 7 participants from the focus groups further volunteered to guide the development of the video game by forming a Board of Advisors that oversaw the writing of game dialogue and creation of character storylines, as well as provided feedback on character art and design. The characters and background images in *InvestiDate* were designed and illustrated by a young Black female graphic artist. The educational content of the intervention game was determined through the examination of other HIV and STI prevention programs, existing literature, and through the creation of a Game Playbook [39] guided by theories of behavior change [40,41]. Game development was achieved through collaboration with professional educational game developers. A typical game takes between 30 and 45 minutes to complete. Participants in the intervention condition played 1 to 2 games during each gameplay session, resulting in 70-130 minutes of total intervention exposure.

#### Game Development

*InvestiDate* is based on a theory-driven and evidenced-informed HIV and STI prevention social card game designed for young Black women [37]. The video game adaptation was developed in collaboration with PreviewLabs, Inc, a rapid prototyping and game development company located in New Haven, Connecticut, United States. The video game intervention is a multiplayer and feminist take on a traditional dating simulation (sim) game and is dialogue based, containing no explicit sexual imagery or descriptions. Dating sim games are typically dialogue heavy and involve the building and maintenance of a relationship with one or more characters, with game outcomes largely dependent on statistics. The intervention game adds a social component to the dating sim genre by pitting players against one another in a race to develop relationships with the datable characters. Furthermore, an element of icebreaker games is added in that players are encouraged to predict each other’s reactions to game events. The intervention game also borrows structural components form the hit social game series Jackbox, in that game action takes place on a single shared screen, which runs on desktops and laptops with Windows (Microsoft Corp) or Mac (Apple Inc) operating systems while players input gameplay decisions on separate internet-capable devices through a web-based interface.

#### Theoretical Underpinnings

**Figure 2:**
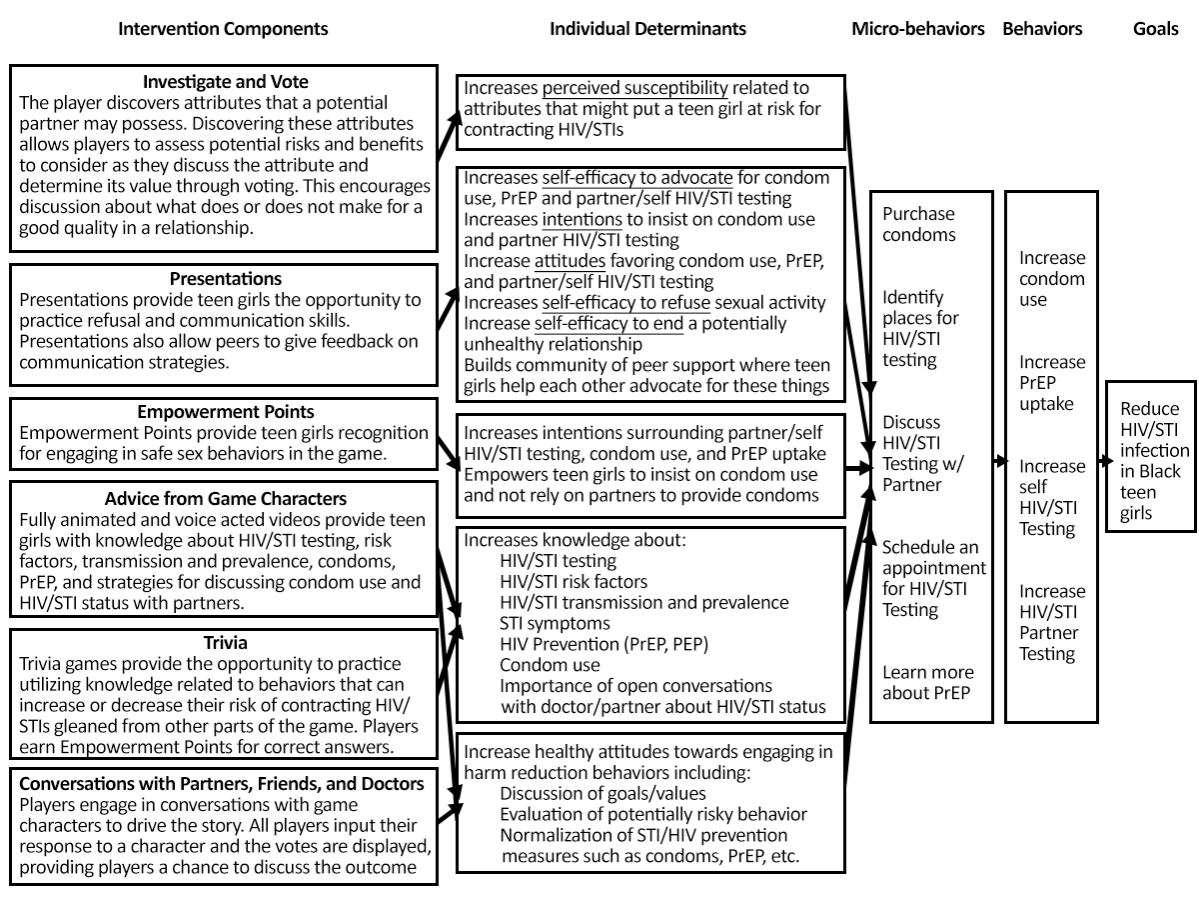
Game intervention conceptual model. PrEP: pre-exposure prophylaxis; STI: sexually transmitted infection.

The conceptual model of the game intervention depicted in Figure 2 is based on well-established behavior change theories, including social cognitive theory [40] and health belief model [42]. Social cognitive theory [43,44] posits that behavior change occurs through the development of self-efficacy and skill acquisition and that learning is expedited through the observation of behavior carried out by others similar to the observer. Self-efficacy can be achieved through behavioral rehearsal and feedback, and the observation of behavior is simulated through the inclusion of roleplaying storylines based on the lived experiences of Black adolescent girls who participated in the formative focus groups. The health belief model posits that if a person perceives a benefit from a particular change in behavior, they will be more likely to adhere to the change [42]. In the video game, players earn points for behaviors like testing for HIV and STI, insisting on condom use, and communicating with partners extensively. These vicarious learning opportunities model beneficial behavior for players.

#### Gameplay

**Figure 3:**
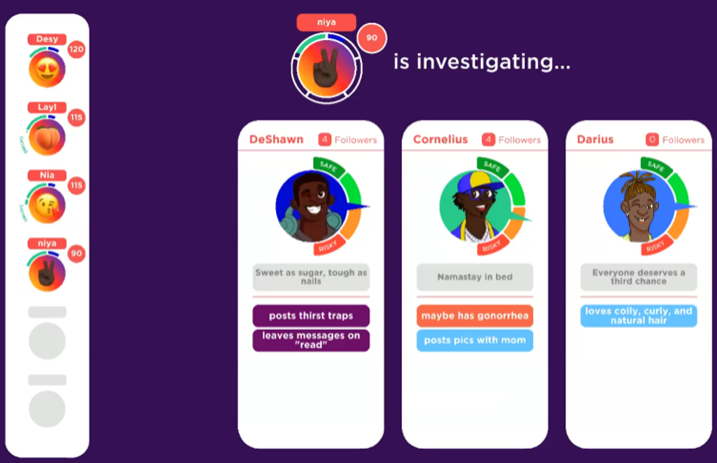
Players, represented by emojis on the left of the screen, choose whom to “follow” based on biography and profile image and whom to investigate based on traits revealed on previous turns.

**Figure 4:**
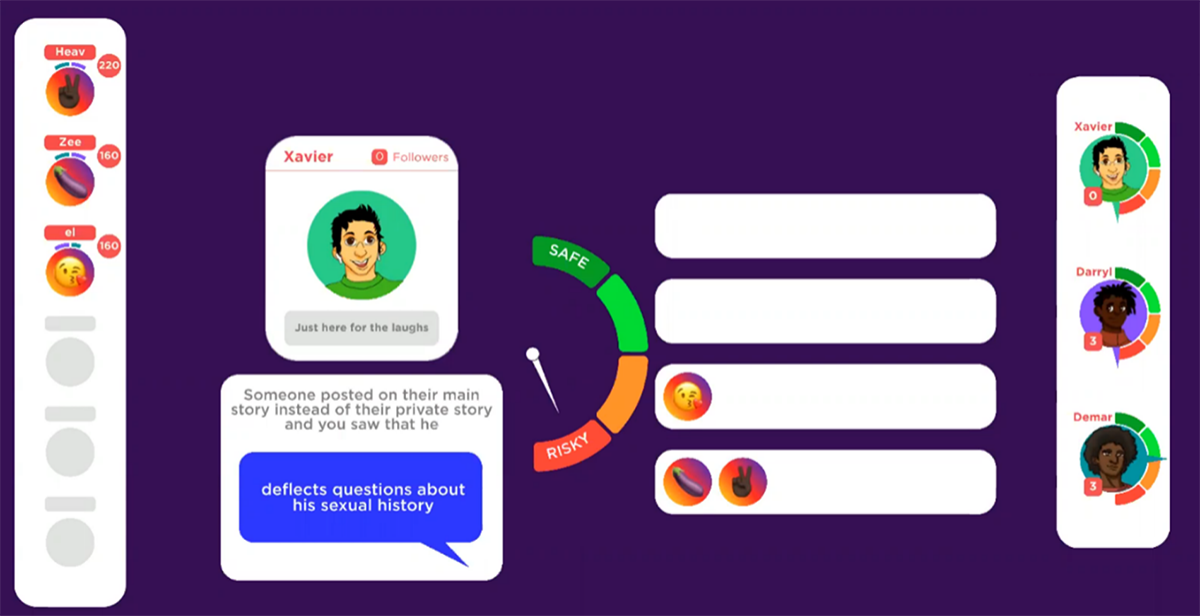
Players investigate datable characters and then vote on traits on a scale from safe-risky (pictured below) and cool-lame. Dating more risky characters increases the likelihood of a player becoming diagnosed with HIV or another sexually transmitted infection (STI).

**Figure 5:**
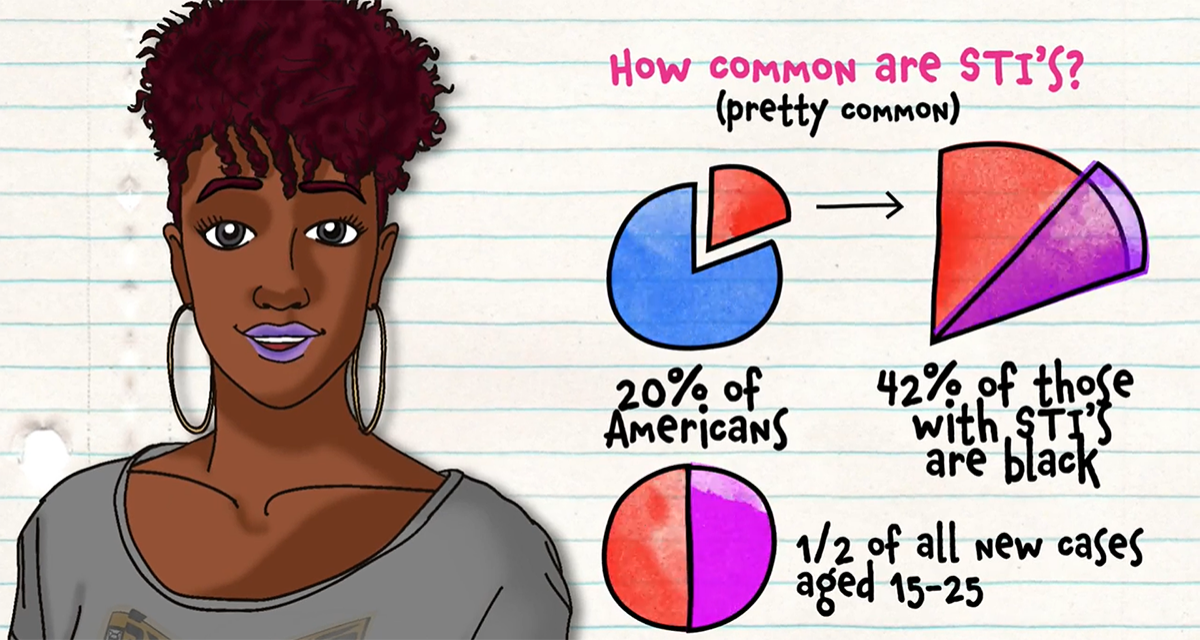
The character Chelsea explains sexually transmitted infection (STI) prevalence in the United States in an animated voice-acted video.

**Figure 6:**
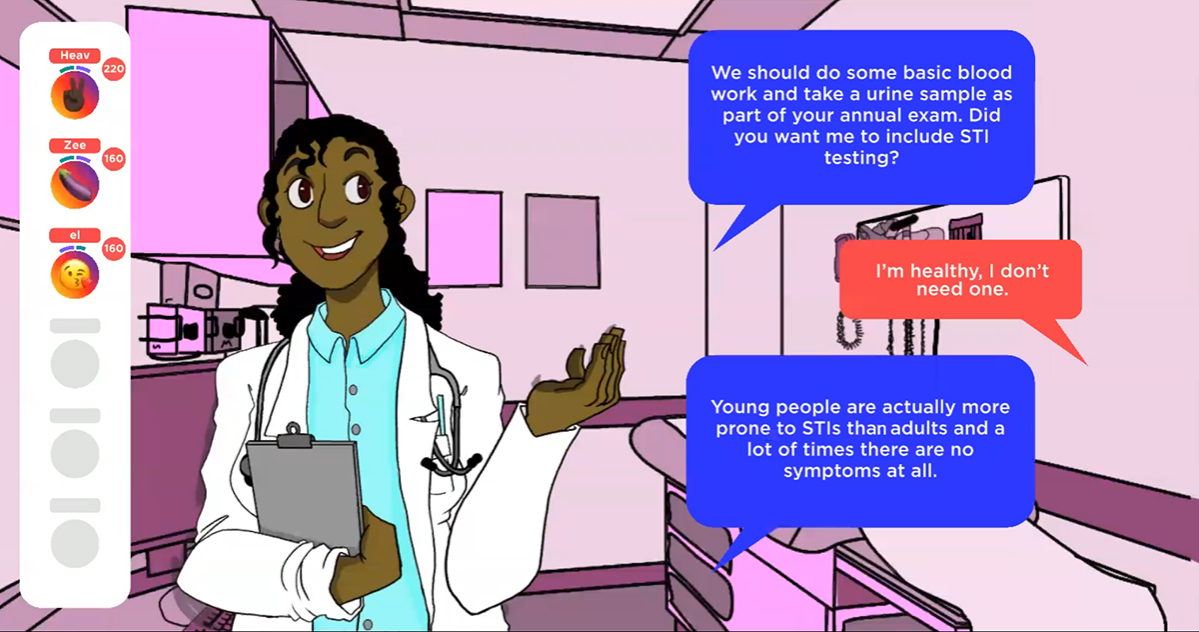
A conversation about HIV and sexually transmitted infection (STI) testing with a doctor.

At the beginning of the game, the players are introduced to “Chelsea” and “Brandon,” 2 nonplayable characters who serve as guides throughout the game by providing instructions and verbal prompts to capture and direct player attention. In addition to learning a bit about the 2 host characters, all the players must pick 5 goals from options such as “talk to your doctor about PrEP,” “convince a guy to use a condom during sex,” “answer 9 trivia questions correctly,” and “get tested for HIV and STIs,” all with varying amounts of points awarded for completion. The game concludes when the first player reaches 300 points, and goal achievement provides the highest point awards. Figures 3-6 depict screenshots from the game and provide examples of actions players can take during the game to achieve goals.

### Data Collection

Data were collected in 2 unique ways. First, all the assessments were self-administered via Qualtrics, which allows participants to complete assessments on their computer, phone, or tablet. Participants completed the baseline and postgameplay assessments during a live Zoom session with the researcher, which ensured that participants could ask questions of the study personnel as they arose. The 1-month and 4-month follow-up assessments were emailed to participants and completed by participants on their own time. Second, recordings of all gameplay sessions allow for the transcription of game content and player interactions during the game for qualitative analysis.

### Outcomes

#### Primary Outcomes

The primary outcome of this study is to evaluate the feasibility and acceptability of a multiplayer game–based intervention designed to increase HIV and STI testing and condom use among Black adolescent girls. Intervention feasibility and acceptability will be examined through the number of sessions completed by participants and participant retention rates throughout the study.

#### Secondary Outcomes

Our secondary outcome is to increase Black adolescent girls’ HIV and STI testing; use of condoms; self-efficacy to use condoms; positive attitudes toward condom use; intentions to use condoms; harm perceptions; HIV, STI, and pre-exposure prophylaxis (PrEP) knowledge; positive sexual norms; sexual communication with partners; and reduced incidence of sexual risk behaviors associated with HIV and STI transmission. We hypothesize that participants in the intervention condition will demonstrate greater improvements in the above outcomes when compared with the control participants. We will also measure whether changes are sustained across the follow-up periods of 1- and 4 months, as well as intervention feasibility and acceptability. Table 1 lists the surveys used to evaluate our secondary aim.

Across all time points, HIV and STI knowledge is evaluated with 12 true or false statements such as “having an STI like herpes can put a person at greater risk for getting HIV” or “chlamydia and gonorrhea cannot be cured,” and recoded as correct or incorrect. The 12-item assessment is based on the validated 45- and 27-item HIV- and STI-Knowledge Questionnaires [45,46] and is available in Multimedia Appendix 1 [45,46]. Participants have the option to select “I don’t know,” which is scored as incorrect. A cumulative knowledge score will be created for each participant, with a maximum score of 12. CUSES (Condom Use Self-Efficacy Scale) is measured with a 10-item adaption of the scale designed by Brafford and Beck [47] and includes questions like “If my partner didn’t want to use a condom, I feel confident that I could convince him that it was necessary” and “I feel confident in my ability to put a condom on my partner” to which participants could respond 1 (strongly disagree) to 5 (strongly agree). SRBBS (Sexual Risk Behavior Beliefs and Self-efficacy) is measured with a 27-item Likert scale questionnaire that includes questions like “Most of my friends believe people my age should wait until they are older before they have sex” or “If you decided to have sex, how sure are you that you could have a condom with you when you needed it?” to which participants respond on a 5-point scale [48]. PrEP knowledge and attitudes were assessed with questions regarding participant familiarity with PrEP, whether they would approve of friends or partners taking PrEP, and whether they believed friends and partners would approve if they were to take PrEP. Finally, sexual risk behavior was assessed with self-report questions based on the Youth Risk Behavior Survey [49]. We hypothesize that participants in the intervention condition will have greater changes or improvements in the above outcomes when compared with the control participants and that these changes will be sustained across time points.

#### Exploratory Outcomes

An exploratory outcome is included to evaluate participants’ real-time responses to the game through the content analysis of audio recordings of gameplay sessions. We will investigate participant perceptions of research procedures and game content to analyze suggestions for the improvement of both. An additional exploratory analysis seeks to determine whether there is a relationship between participant responses on the 6-item MEIM-R (Multigroup Ethnic Identity Measure—Revised) and other outcome measures. The connection between ethnic identity and self-esteem, for example, is well known [50]. We will explore whether connection to one’s ethnic identity may be a protective factor against sexual risk behavior [38]. Because the exploratory aim concerns implementation procedures and the adaptation and expansion of the game, there are no a priori hypotheses.

### Statistical Analysis

All statistical analyses will be conducted on an intention-to-treat sample using SPSS Statistics (IBM Corp) packages. Baseline characteristics will be analyzed using descriptive statistics. We will use baseline, postgameplay, 1-month, and 4-month data to estimate effect sizes for the primary outcomes. We will test the hypothesis that individuals who play *InvestiDate* will report more HIV and STI testing and communication with partners about testing in comparison to the control condition. We will conduct a longitudinal analysis using a hierarchical linear mixed models approach to compare participants in the intervention group to participants in the control group on reported participant HIV and STI testing from baseline across all follow-up assessments (ie, baseline, 1 month, and 4 months). Hierarchical modeling will allow us to account for the fact that the data are nested, as the participants will play the game in groups. An advantage of mixed models is that participants with missing data need not be excluded as all available data are used in estimating parameters. We will also use logistic regression models to determine if any relevant baseline variables, including ethnic identity, are associated with improvement in reported HIV and STI testing (constructed as a binary outcome [yes or no]). We plan to evaluate the prognostic significance of a small set of predictor variables (eg, attitudes, self-efficacy, ethnic identity, and knowledge) and use them as covariates in the primary analysis. Given the limited sample size (n=79), we anticipate that the power of this study to identify small but significant effect sizes will be low. A strength of this design is that it provides a number of perspectives from which to view treatment effects, including a treatment condition, a control condition, and a follow-up period. The results will inform the sample size calculation for a subsequent larger RCT.

### Qualitative Analysis

The transcripts and chat logs from *InvestiDate* gameplay sessions (experimental group only) will be reviewed by members of the research team jointly who will perform data coding. These codes will be created in a step-wise fashion [51], beginning with the creation of an initial code structure from the first 2 transcripts that are independently reviewed by each team member. To enhance interrater reliability, gameplay transcripts will be independently reviewed and coded by each researcher and coding will be compared for agreement. Manual coding will then be electronically applied to the textual data using Dedoose (SocioCultural Research Consultants), a software program designed to facilitate the analysis of qualitative data. Data collected will be used to inform subsequent iterations of the video game, *InvestiDate*, and will similarly be used to provide exploratory evidence for the success of the intervention at achieving primary outcomes such as normalization of conversations about sex and safe sex practices such as testing and condom use.

## Results

### Enrollment and Randomization

**Figure 7:**
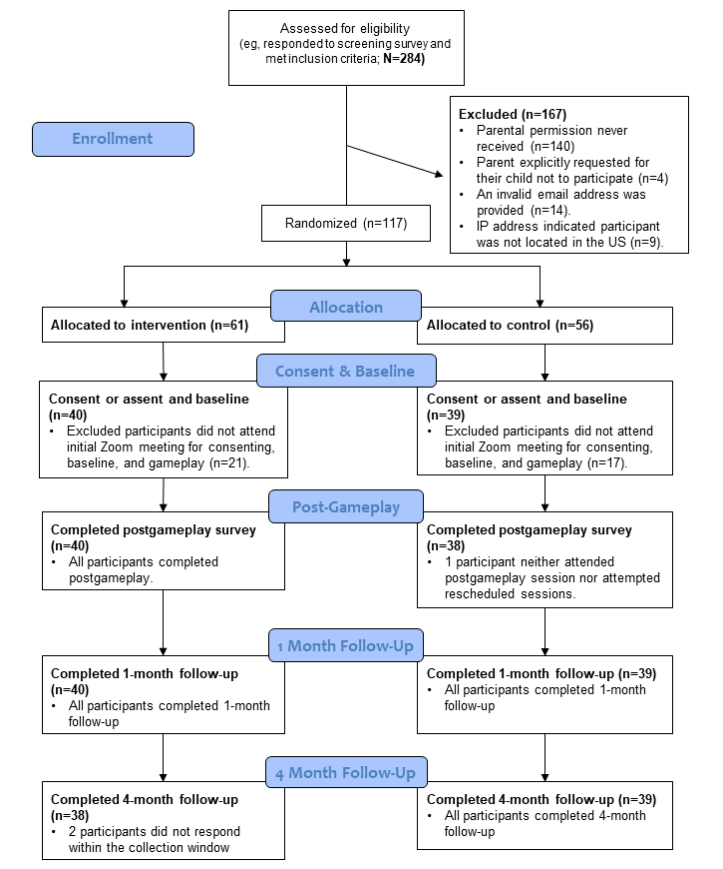
CONSORT (Consolidated Standards of Reporting Trials) flow diagram depicting participant enrollment and retention across all study time points. US: United States.

Overall, 1497 individuals clicked the link to the screening survey. In total, 47.43% (710/1497) of individuals completed the screening questionnaire, 40% (284/710) of which met the eligibility criteria; 9.5% (27/284) of eligible respondents were 18 years old at the time of recruitment, and we received parental consent for an additional 31.7% (90/284) of individuals to participate. Of those 117 prospective participants, 79 (67.5%) girls attended the first gameplay session, consented to participate, and were enrolled. Subsequently, 51% (40/79) of the enrolled participants were randomized into the intervention condition, and 49% (39/79) of the participants into the control condition. Figure 7 shows the CONSORT (Consolidated Standards of Reporting Trials) flow diagram that depicts participant retention at all time points [52]. All 79 participants were enrolled into the RCT between February 2022 and April 2022. All 39 participants in the control condition and 38 participants in the intervention condition completed the final survey in September 2022.

### Sample Demographics

At baseline, there were no significant differences in demographic characteristics between intervention and control condition participants. At baseline, participant ages ranged from 14 to 19 years (mean 16.4, SD 1.23). One participant turned 19 during the week that elapsed between recruitment and the first gameplay session. Although the screening survey excluded participants who identified as having a sexual orientation other than straight (ie, heterosexual), bisexual, or pansexual, at baseline 10.1% (8/79) of participants identified as questioning or unsure, 1.2% (1/79) as queer, and 1.2% (1/79) as asexual, with the remaining 55.6% (44/79) of the participants identifying as straight or heterosexual and 32.9% (26/79) of the participants as bisexual or pansexual.

## Discussion

### Overview

In this pilot RCT of a multiplayer game–based intervention designed to increase HIV and STI testing and condom use among adolescent Black girls, we enrolled and randomized 79 participants. All participants were recruited through Instagram or Facebook, and there were no significant demographic differences between participants randomized to the intervention and control conditions. Notably, we chose to expand our inclusion criteria from strictly heterosexual-identifying participants to include individuals who identified as bisexual or pansexual in response to high numbers of participants who self-identified as something other than heterosexual. This observation is consistent with recent data from the Adolescent Behaviors and Experiences Survey in which 1 in 5 high schoolers identified as something other than straight or heterosexual [53].

This study protocol offers several strengths and novel strategies that can inform future gender- and culturally tailored HIV and STI prevention interventions as well as inform efforts to collect data for RCTs remotely using extant platforms like Zoom. Our video game intervention specifically addresses the intersectional identities of Black adolescent girls by incorporating the lived experiences of focus group participants into game story lines. In addition, the video game fosters a sense of social support by allowing Black adolescent girls to interact with each other, which is an important protective factor for sexual health [38]. It is among the first targeted interventions that is sex positive and harnesses the power of peer interactions through multiplayer gameplay to deliver HIV and STI prevention material. Because the video game intervention is self-contained, it has the potential to overcome common barriers to existing HIV prevention interventions, including access to adequately trained providers and intervention fidelity. Finally, data were successfully collected remotely during the global COVID-19 pandemic when in-person interactions were restricted. The procedures developed for data collection in this intervention can be adopted and used in other web-based studies involving multiple synchronous participants and may also be useful for the collection of larger nationally distributed samples.

### Limitations

There are some limitations to this study protocol. In particular, participants were conveniently sampled through social media advertising, which may have resulted in selection bias. To participate in the study, participants were required to be available for gameplay sessions during weekday evenings, have a strong internet connection, and had to have access to 2 separate internet-capable devices (one to use the Zoom platform and one to play the game). These requirements may have discouraged participants with full evening schedules such as afterschool activities, part-time jobs, or family responsibilities from participating, as well as participants with low socioeconomic status. Finally, although the game was intended to be a social experience, game audio (such as background music and video game character dialogue) made communication between players difficult during many game sequences. Decreased social interaction in the multiplayer game may impact both participant ratings of gameplay satisfaction and acceptability, as well as intervention outcomes. In future iterations, we will examine whether in-person gameplay is more satisfactory than remote play over Zoom.

### Conclusions

Black adolescent girls have the highest rates of STI among ethnic minority girls and are more than twice as likely to be diagnosed with an STI than their White peers, but there are very few HIV and STI prevention interventions specifically tailored for this group. This paper describes our protocol for a pilot RCT of a multiplayer game–based intervention designed to increase HIV and STI testing and condom use in Black adolescent girls. The aims of this pilot study are to assess the feasibility, acceptability, and preliminary efficacy of the game intervention. This protocol directly informs the development of a future larger RCT testing the efficacy of the video game intervention, *InvestiDate*, and can serve as an example for other researchers seeking to collect data over Zoom from multiple synchronous participants distributed nationally.

## Appendix

Multimedia Appendix 1 A 12-item assessment based on the validated 45- and 27-item HIV and sexually transmitted infection (STI) Knowledge Questionnaires.

Multimedia Appendix 2 Peer review report by Behavioral and Social Science Approaches to Preventing HIV/AIDS (BSPH) Study Section AIDS and Related Research Integrated Review Group Center for Scientific Review (National Institutes of Health, USA).

## Abbreviations

CONSORT: Consolidated Standards of Reporting Trials
CUSES: Condom Use Self-Efficacy Scale
MEIM-R: Multigroup Ethnic Identity Measure—Revised
PrEP: pre-exposure prophylaxis
RCT: randomized controlled trial
SRBBS: Sexual Risk Behavior Beliefs and Self-efficacy
STI: sexually transmitted infection

## Data Availability

The data generated during this study will be available from the corresponding author on reasonable request.
