# Supplementary material for: Pilot-testing a Multiplayer HIV and Sexually Transmitted Infection Prevention Video Game Intervention for Black Adolescent Girls: Protocol for a Randomized Controlled Trial
Source: JMIR Res Protoc. 2023 Jan 23;12:e43666. doi: 10.2196/43666 (PMC9903186; doi:10.2196/43666)
Supplement: Multimedia Appendix 1 [file resprot_v12i1e43666_app1.pdf]

Appendix 1: 12-item HIV/STI Knowledge Questionnaire

1. Herpes can be cured with antibiotics

- ○ True
- ○ False
- ○ Not sure

---

2. Having an STI like herpes can put a person at greater risk for getting HIV

- ○ True
- ○ False
- ○ Not sure

---

3. You can tell if someone has an STI, including HIV, just by looking at them

- ○ True
- ○ False
- ○ Not sure

---

4. If you have gonorrhea but do not have any symptoms, you can still spread it to your sexual partners

- ○ True
- ○ False
- ○ Not sure

---

5. Chlamydia and gonorrhea cannot be cured

- ○ True
- ○ False
- ○ Not sure

---

6. Condoms can prevent pregnancy but cannot prevent the spread of HIV

- ○ True
- ○ False
- ○ Not sure

---

7. Sexual contact is the most common way that HIV infections occur

- ○ True
- ○ False
- ○ Not sure

---

8. It is possible to contract HIV from just one unprotected sexual encounter

- ○ True
- ○ False
- ○ Not Sure

---

9. People who have more sexual partners are more at risk for HIV or STIs

- ○ True
- ○ False
- ○ Not sure

---

10. Untreated chlamydia can cause permanent damage to a woman's reproductive system

- ○ True
- ○ False
- ○ Not sure

---

11. People who have been infected with HIV quickly show serious signs of being infected

- ○ True
- ○ False
- ○ Not sure

---

12. There is a cure for syphilis

- ○ True
- ○ False
- ○ Not sure
