# Supplementary material for: Pilot-testing a Multiplayer HIV and Sexually Transmitted Infection Prevention Video Game Intervention for Black Adolescent Girls: Protocol for a Randomized Controlled Trial
Source: JMIR Res Protoc. 2023 Jan 23;12:e43666. doi: 10.2196/43666 (PMC9903186; doi:10.2196/43666)
Supplement: Multimedia Appendix 2 [file resprot_v12i1e43666_app2.pdf]

SUMMARY STATEMENT
(Privileged Communication)

PROGRAM CONTACT:
Denise Russo
301-435-6871
drusso1@mail.nih.gov

Release Date: **08/06/2018**
Revised Date:

Application Number: **1 R21 HD098031-01**

Principal Investigator

HIEFTJE, KIMBERLY D

Applicant Organization: YALE UNIVERSITY

Review Group: **BSPH**
Behavioral and Social Science Approaches to Preventing HIV/AIDS Study Section
AIDS - EXP. REV.

Meeting Date: **07/11/2018**
Council: **OCT 2018**
Requested Start: **12/01/2018**
RFA/PA: **PA18-482**
PCC: **MPIDB-DR**

Project Title: **A Digital Intervention for HIV Prevention in Black Adolescent Girls**

SRG Action: **Impact Score:40 Percentile:20 +**
Next Steps: **Visit https://grants.nih.gov/grants/next_steps.htm**
Human Subjects: 30-Human subjects involved - Certified, no SRG concerns
Animal Subjects: 10-No live vertebrate animals involved for competing appl.
Gender: 2A-Only women, scientifically acceptable
Minority: 2A-Only minorities, scientifically acceptable
Children: 1A-Both Children and Adults, scientifically acceptable

Project Year	Direct Costs Requested	Estimated Total Cost
1	125,000	209,375
2	150,000	251,250
TOTAL	275,000	460,625

ADMINISTRATIVE BUDGET NOTE: The budget shown is the requested budget and has not been adjusted to reflect any recommendations made by reviewers. If an award is planned, the costs will be calculated by Institute grants management staff based on the recommendations outlined below in the COMMITTEE BUDGET RECOMMENDATIONS section.

1R21HD098031-01 Hieftje, Kimberly

RESUME AND SUMMARY OF DISCUSSION: The applicant proposes to evaluate the feasibility and preliminary efficacy of an adaptation of “One Night Stan,” originally a card game designed to increase partner HIV/STI testing and condom use in young Black women aged 18- 24. The current (adapted) multiplayer videogame, is designed to promote self and partner HIV/STI testing for young Black adolescent girls ages 14-18. The intervention’s impact on increasing players’ HIV/STI knowledge and for increasing intentions and attitudes around safer HIV/STI practices will also be explored. The premise of this application is very strong, young Black girls are bearing a disproportionate burden of HIV; they are often unaware of their partners serostatus and engage in unprotected sex. The adaptation of a card game into a videogame accessible on a smartphone is a strength of this application as it uses technology with which this population is familiar and likes to use, and is scalable The implementation of a multiplayer videogame, and the use of an iterative user-centered design to modify the intervention make for an innovative application. This investigator is experienced in developing and researching eHealth gaming; his collaborators are likewise accomplished, having the complementary skills that ensure the success of this project. The methodology and analyses of this application were generally rigorous, including such approaches as a sequential focus group feedback to the matched control group. The committee was divided about the merit of the application with some reviewers judging it as potentially having high impact and others a moderate impact at best. The weaknesses raised that dampened enthusiasm for the study were the following: it is unclear why One Night Stan was transformed from a card game to a videogame; no justification was presented for that; no preliminary efficacy data. The testing of partners is one of the primary outcomes of this study, yet some girls may not have partners; most of the outcome measures are self-reports that lend themselves to social desirability; moreover, no protocol has been developed to validate those self-reports; there is a focus on a population of Black girls who are already engaged in services and afterschool programs; this may yield a biased sample since most at-risk young women would likely not be recruited. Despite these weaknesses, this application’s strengths surpass its weaknesses and most of the committee believes that the application stands to make a contribution to the field in promoting self-testing and partner testing among young black girls.

DESCRIPTION (provided by applicant): Black women are disproportionately diagnosed with human immunodeficiency virus (HIV) and sexually transmitted infections (STIs). Black women often contract HIV/STIs through high-risk sex and adolescence is when these behaviors are more common. Furthermore, Black adolescent girls are more affected by HIV/STIs than other demographics of adolescent girls; yet, there are few engaging and effective interventions designed exclusively for them. In fact, many are unaware of their partner HIV/STI status and do not consistently use condoms to protect themselves. Therefore, interventions that counter individual-level risk perception by promoting HIV/STI testing for themselves and their partners and reduce risk behaviors, such as non-condom use, are greatly needed. There is compelling evidence that peers play a significant role in the protection from risk associated with HIV/STIs by providing opportunities to exchange knowledge, influence attitudes, and to develop new behavioral skills. Videogame interventions, which are frequently played with others, can be culturally tailored, provide behavioral skills training, and have demonstrated efficacy in affecting health behaviors related to health promotion. With the growing evidence for the efficacy of videogames in improving health outcomes, it is essential to examine how to harness these technologies to modify behaviors in Black adolescent girls. With funding from the Women’s Health Research at Yale Pilot Program, the Yale’s play2PREVENT Lab developed and pilot tested a theory-based social card game intervention, One Night Stan, which was aimed at increasing partner HIV/STI testing and condom use in young black women aged 18- 24. Preliminary findings of a pilot study conducted with 21 black women aged 18-21 suggest that playing the game can lead to increased self-efficacy and intentions to use condoms and insist that their partners get tested for HIV across time. In terms of gameplay satisfaction, 100% of participants reported that that they would play the game again, 95% liked the way the game looked, 100% enjoyed playing the game, and 100% reported that they would tell their friends

to play. Results from this study suggest that One Night Stan is a feasible intervention approach and may be efficacious in helping players develop a pattern of cognitions and motivation that can protect them against the risk of HIV. Together, a team comprised of experts from the Yale Center for Health & Leaning Games will build upon this formative work and pilot data to adapt One Night Stan to a multiplayer videogame for younger target audience of Black adolescent girls. For this proposal, we will evaluate the feasibility and preliminary efficacy of One Night Stan at increasing self and partner HIV/STI testing by conducting a pilot randomized controlled trial with 80 young Black adolescent girls ages 14-18. We will also explore the efficacy of the intervention for increasing players' HIV/STI knowledge and for increasing intentions and attitudes around safer HIV/STI practices. As a multiplayer videogame, the intervention is a compelling framework for Black adolescent girls who constantly interact and are influenced by their peers.

PUBLIC HEALTH RELEVANCE: This project is designed to develop and test a multiplayer videogame intervention for adolescent black girls ages 14-18. The goal of this proposal is to increase HIV/STI self and partner testing and reduce sexual risk behaviors such as non-condom use in order to decrease their risk for human immunodeficiency virus (HIV) and other sexually transmitted infections (STI). Because the game will be played with adolescent females and their peers, this project has the potential to spread beyond the individual to influence public health and decrease the number of new cases of HIV/STI.

CRITIQUE 1

Significance: 3
Investigator(s): 2
Innovation: 4
Approach: 5
Environment: 2

Overall Impact: The overall goal of this R21 application is to develop and pilot test a group-based game for Black adolescent girls (ages 14-18) who are sexually active. The scientific premise of the study is strong, as there is a need to curtail HIV disparities among this group. The team is well-equipped to carry out the proposed study, as they have prior expertise in eHealth gaming. Overall, the proposed intervention is innovative in its mode of delivery, yet the overall impact of the intervention is diminished by concerns regarding sample selection, empirical justification for migrating the card game into a videogame format, and issues regarding the pilot trial design.

1. Significance:

Strengths

- Young women, particularly racial/ethnic minority women, remain a high priority population for HIV prevention.
- eHealth games can promote behavior change and have greater potential for scalability if found to be efficacious.

Weaknesses

- Unclear why team decided to make game playable only in person, as this approach counters the benefits of an online game delivery modality.

- Limited justification for the transition from a card game (One Night Stan) into a game. There is no preliminary efficacy provided; furthermore, it seems the original game was built for an older population than the one being studied in this application.

2. Investigator(s):

Strengths

- PI has expertise conducting e-health interventions for youth.
- Co-I team is strong, with complementary expertise.

Weaknesses

- None Noted

3. Innovation:

Strengths

- The use of multiplayer gaming to deliver an intervention is innovative.
- Iterative revisions based on user-centered feedback is noteworthy.

Weaknesses

- The use of qualitative data to understand Black teenagers' risk behaviors is not innovative and has been explored thoroughly in the literature.

4. Approach:

Strengths

- Sequential focus group feedback is a strength of the application as the team integrates feedback on the game design and logistics.
- Peer-to-peer feedback during game design is a strength of the approach.

Weaknesses

- It is unclear what the intervention components are, and how they align with developmental considerations.
- The team proposes a grounded theory approach in Phase 1, which seems inadequate for the proposed work and the study goals.
- Why is partner testing a primary outcome of the study? What if a participant doesn't have a partner? Given the pilot nature of the study, I would focus solely on participants' behaviors (e.g., HIV/STI testing, sex risk).
- Since groups will be kept consistent after randomization, how do you know whether the game creates the expected intervention effect or if it's a result of group bonding? Further, it would seem like adjusting for the clustering effect may be needed.
- The control condition is not matched to the intervention arm, reducing the rigor of the proposed approach.
- Satisfaction/acceptability should be asked in both arms.
- Power estimates should include proposed effect sizes.

5. Environment:

Strengths

- Yale has the necessary resources to support the proposed work.

Weaknesses

- None Noted

Study Timeline:

Strengths

- Timeline provided seems feasible, with sufficient cushion to overcome any potential delays that may arise during the project start-up and programming phase.

Weaknesses

- None Noted

Protections for Human Subjects:

Acceptable Risks and/or Adequate Protections

- Team should consider how to circumvent potential contamination due to peer exchanges and intended/unintended disclosure by peers.
- Strongly encourage team to explore a waiver of parental consent to avoid unintended disclosure of sexual activity to family, and reduce selection bias during recruitment.

Data and Safety Monitoring Plan (Applicable for Clinical Trials Only):

Acceptable

- DSMP is adequate and will be overseen by Yale

Inclusion of Women, Minorities and Children:

- Sex/Gender: Distribution justified scientifically
- Race/Ethnicity: Distribution justified scientifically
- For NIH-Defined Phase III trials, Plans for valid design and analysis: Not applicable
- Inclusion/Exclusion of Children under 18: Including ages <18; justified scientifically
- Black/AA young women (14-18 years) will be enrolled; justified.

Vertebrate Animals:

Not Applicable (No Vertebrate Animals)

Biohazards:

Not Applicable (No Biohazards)

Resource Sharing Plans:

Acceptable

Authentication of Key Biological and/or Chemical Resources:
Not Applicable (No Relevant Resources)

Budget and Period of Support:
Recommend as Requested:

CRITIQUE 2

Significance: 2
Investigator(s): 2
Innovation: 2
Approach: 2
Environment: 1

Overall Impact: This is an innovated new R21 application that seeks to develop and test a multiplayer videogame intervention for black adolescent and young women (BAYW) using cellphones based on a prior developed multi-player card game "One Night Stan". There is a clear need for interventions for this population around issues of HIV and STI prevention and a videogame-based intervention could be highly scalable and culturally adaptable to other populations and languages. The scientific premise of the use of informational games to increase knowledge and intentions for health behaviors is established in the literature and the prior testing of the card game also supports this premise. The team is very qualified, if somewhat junior, to carry out the research and has a prior history of videogame development and testing. The approach is rigorous and well structured. Minor weaknesses of the study are a reliance on self-reported outcome measures and recruitment of youth who may already be engaged in services which may exclude some high-risk girls and women. Overall an excellent "high-risk, high-reward" R21 proposal that could have significant reach and impact.

1. Significance:
Strengths
- Black adolescent and young adult women are at increased risk of HIV and disparities in HIV testing and treatment continue to exist.
- New channels to engage and impact this population are needed and video games present a significant opportunity given the frequency of use among youth.
- Preliminary work on "One Night Stan" enhances the feasibility of the proposed research and supports the intervention's scientific premise.
- Videogame-based intervention could be highly scalable – and culturally adaptable to other populations and languages.
- Group has successfully developed and tested a related videogame for at-risk boys and girls ages 11-14, providing further feasibility and capability of the proposal.
- Preliminary impact data will be very useful in comparative efficacy trial planning.

Weaknesses

- Marketing and uptake for an HIV/STI based game will potentially face challenges of stigma and appeal.
- Few objective endpoint measures will be examined and reliance on self-report.

2. Investigator(s):

Strengths

- PI is published in the areas of HIV, youth, and game development.
- Co-I is Assoc. Director of play2PREVENT lab at Yale.
- Team has expertise in health behavior psychology, game development, HIV/AIDS prevention.
- Co-I Fiellin has experience with current and prior NIH grants.

Weaknesses

- PI does not have prior NIH grant leadership experience and is somewhat junior.

3. Innovation:

Strengths

- Use of a multi-player videogame as an intervention for BAYW is highly innovative.
- Inclusion of peer element in the context of game play is novel.

Weaknesses

- Qualitative research plans cited are not really an innovation.
- It would have been nice to see some innovation in methods of outcome assessments

4. Approach:

Strengths

- Strong intervention development plan and a history of previous successful development and testing of a videogame intervention.
- Demonstrated capacity for proposed recruitment methods to attract and enroll subjects.
- Focus group plans and analysis are good.
- Use of smartphones as controllers/platform is highly desirable.
- Matched control condition is rigorous.
- Methods for retention and follow-up are strong.
- Phase-2 development plan is clear and web-platform for game is feasible.
- Assessment scale are validated instruments.
- Longitudinal follow-up is a strength.
- Analysis plans are clear and appropriate.

Weaknesses

- Study population focuses on BAYW already engaged in services and afterschool programs and may miss out on high-risk unengaged persons.

- Exclusion of those with HIV testing in the last year may bias subject towards lower risk groups. Suggest eliminating this criterion, as more frequent HIV testing for those with risk is advisable.
- Phase 2 testing mandates peer players be others in the study and not necessarily their true social network/peers.
- Sample size estimation and/or level of confidence on estimates are not provided.
- No methods are provided for assessing and validating self-report of HIV/STI and partner HIV/STI testing.

5. Environment:

Strengths

- Yale University and its associated CFAR have a significant history of supporting successful HIV research.
- Association with PreviewLabs is ongoing and has significant relevant experience and productivity.
- Letters of support from recruitment sites.

Weaknesses

- None Noted

Study Timeline:

Strengths

- Reasonable timelines for Phases 1 & 2 and game development and testing

Weaknesses

- None Noted

Protections for Human Subjects:

Acceptable Risks and/or Adequate Protections

- Acceptable

Data and Safety Monitoring Plan (Applicable for Clinical Trials Only):

Acceptable

- Minimal Risk

Inclusion of Women, Minorities and Children:

- Sex/Gender: Distribution justified scientifically
- Race/Ethnicity: Distribution justified scientifically
- For NIH-Defined Phase III trials, Plans for valid design and analysis: Not applicable
- Inclusion/Exclusion of Children under 18: Including ages <18; justified scientifically
- Acceptable

Vertebrate Animals:

Not Applicable (No Vertebrate Animals)

Biohazards:

Not Applicable (No Biohazards)

Authentication of Key Biological and/or Chemical Resources:

Not Applicable (No Relevant Resources)

Budget and Period of Support:

Recommend as Requested:

CRITIQUE 3

Significance: 1
Investigator(s): 1
Innovation: 1
Approach: 5
Environment: 1

Overall Impact: The proposed work aims to develop and conduct a preliminary test of a social videogame to increase HIV/STI testing and condom use among Black adolescent girls, a population that is disproportionately affected by HIV/STIs. The scientific premise for the proposed work is strong. The proposed project builds on a promising card game developed for this purpose. The investigative team is strong. The work is innovative in its use of a social videogame aimed at HIV/STI testing and condom use in this population. The level of scientific rigor is relatively high. The use of a non-health related control condition is a particular concern given the sole reliance on self-report data.

1. Significance:

Strengths

- The scientific premise for the proposed work is strong.
- Young black women and adolescent girls are disproportionately affected by HIV/STIs.
- Social videogames show promise as an effective means of engaging adolescents around health-related messaging.
- The videogame will be based on a promising card game, previously developed and pilot tested by the investigators.

Weaknesses

- None Noted

2. Investigator(s):

Strengths

- The investigative team is strong.
- The team has the necessary and complementary expertise and experience.

Weaknesses

- None Noted

3. Innovation:

Strengths

- The proposed work is innovative in its focus on increasing HIV/STI testing and partner testing and increasing condom use in Black adolescent girls.
- The social aspect of the videogame is also innovative.

Weaknesses

- None Noted

4. Approach:

Strengths

- The level of scientific rigor is relatively high.
- The investigators propose to use a stratified randomization procedure.
- The proposed measures have been validated.
- Sex as a biological variable is not considered given that the study focuses on adolescent girls.

Weaknesses

- The study relies solely on self-report measures.
- The proposed follow-up period of four months is short.
- A non-health related multiplayer videogame does not constitute a credible control condition. This is a particular concern given the reliance on self-reported outcome data. Social desirability effects may be much stronger in the intervention condition. This would be somewhat less of a concern if the control condition focused on health-related content.

5. Environment:

Strengths

- The research environment is strong and has supported similar work in the past.

Weaknesses

- None Noted

Study Timeline:

Strengths

- The proposed timeline appears to be realistic.

Weaknesses

- None Noted

Protections for Human Subjects:
Acceptable Risks and/or Adequate Protections

Data and Safety Monitoring Plan (Applicable for Clinical Trials Only):
Acceptable

Inclusion of Women, Minorities and Children:

- Sex/Gender: Distribution justified scientifically
- Race/Ethnicity: Distribution justified scientifically
- For NIH-Defined Phase III trials, Plans for valid design and analysis: Not applicable
- Inclusion/Exclusion of Children under 18: Including ages <18; justified scientifically

Vertebrate Animals:
Not Applicable (No Vertebrate Animals)

Biohazards:
Not Applicable (No Biohazards)

Resource Sharing Plans:
Unacceptable

- There is no discussion of whether data will be shared with others. There is also no discussion of the availability of the videogame to those outside the study.

Authentication of Key Biological and/or Chemical Resources:
Not Applicable (No Relevant Resources)

Budget and Period of Support:
Recommend as Requested:

THE FOLLOWING SECTIONS WERE PREPARED BY THE SCIENTIFIC REVIEW OFFICER TO SUMMARIZE THE OUTCOME OF DISCUSSIONS OF THE REVIEW COMMITTEE, OR REVIEWERS' WRITTEN CRITIQUES, ON THE FOLLOWING ISSUES:

PROTECTION OF HUMAN SUBJECTS: ACCEPTABLE

INCLUSION OF WOMEN PLAN (G2A): ACCEPTABLE

INCLUSION OF MINORITIES PLAN (M2A): ACCEPTABLE

INCLUSION OF CHILDREN PLAN (C1A): ACCEPTABLE

COMMITTEE BUDGET RECOMMENDATIONS: The budget was recommended as requested.

Footnotes for 1 R21 HD098031-01; PI Name: Hieftje, Kimberly D

+ Derived from the range of percentile values calculated for the study section that reviewed this application.

NIH has modified its policy regarding the receipt of resubmissions (amended applications). See Guide Notice NOT-OD-14-074 at http://grants.nih.gov/grants/guide/notice-files/NOT-OD-14-074.html. The impact/priority score is calculated after discussion of an application by averaging the overall scores (1-9) given by all voting reviewers on the committee and multiplying by 10. The criterion scores are submitted prior to the meeting by the individual reviewers assigned to an application, and are not discussed specifically at the review meeting or calculated into the overall impact score. Some applications also receive a percentile ranking. For details on the review process, see http://grants.nih.gov/grants/peer_review_process.htm#scoring.

MEETING ROSTER

Behavioral and Social Science Approaches to Preventing HIV/AIDS Study Section
AIDS and Related Research Integrated Review Group
CENTER FOR SCIENTIFIC REVIEW
BSPH
07/11/2018 - 07/12/2018

Notice of NIH Policy to All Applicants: Meeting rosters are provided for information purposes only. Applicant investigators and institutional officials must not communicate directly with study section members about an application before or after the review. Failure to observe this policy will create a serious breach of integrity in the peer review process, and may lead to actions outlined in NOT-OD-14-073 at https://grants.nih.gov/grants/guide/notice-files/NOT-OD-14-073.html and NOT-OD-15-106 at https://grants.nih.gov/grants/guide/notice-files/NOT-OD-15-106.html, including removal of the application from immediate review.

CHAIRPERSON(S)

ALLEN, SUSAN A, MD
PROFESSOR
DEPARTMENT OF PATHOLOGY
AND LABORATORY MEDICINE
SCHOOL OF MEDICINE
EMORY UNIVERSITY
ATLANTA, GA 30322

MEMBERS

BARAL, STEFAN DAVID, MD *
ASSOCIATE PROFESSOR
DEPARTMENT OF EPIDEMIOLOGY
BLOOMBERG SCHOOL OF PUBLIC HEALTH
JOHNS HOPKINS UNIVERSITY
BALTIMORE, MD 21205

BAUERMEISTER, JOSE ARTURO, PHD
PROFESSOR
DEPARTMENT OF FAMILY AND COMMUNITY HEALTH
SCHOOL OF NURSING
UNIVERSITY OF PENNSYLVANIA
PHILADELPHIA, PA 19104

BAUMAN, LAURIE J, PHD
PROFESSOR
DEPARTMENT OF PEDIATRICS
ALBERT EINSTEIN COLLEGE OF MEDICINE
BRONX, NY 10461

BLUTHENTHAL, RICKY N, PHD *
PROFESSOR
DEPARTMENT OF PREVENTIVE MEDICINE
KECK SCHOOL OF MEDICINE
UNIVERSITY OF SOUTHERN CALIFORNIA
LOS ANGELES, CA 90033

BOGART, LAURA M, PHD *
SENIOR BEHAVIORAL SCIENTIST
RAND CORPORATION
SANTA MONICA, CA 90407-2138

BROUWER, KIMBERLY C, PHD *
PROFESSOR
DIVISION OF GLOBAL HEALTH
DEPARTMENT OF MEDICINE
SCHOOL OF MEDICINE
UNIVERSITY OF CALIFORNIA, SAN DIEGO
LA JOLLA, CA 92093

CHARLEBOIS, EDWIN DUNCAN, PHD *
PROFESSOR
DEPARTMENT OF MEDICINE
SCHOOL OF MEDICINE
UNIVERSITY OF CALFORNIA, SAN FRANCISCO
SAN FRANCISCO, CA 94105

DICKSON-GOMEZ, JULIA B, PHD
PROFESSOR
CENTER FOR AIDS INTERVENTION RESEARCH
DEPARTMENT OF PSYCHIATRY AND BEHAVIORAL MEDICINE
MEDICAL COLLEGE OF WISCONSIN
MILWAUKEE, WI 53202

DOMBROWSKI, KIRK, PHD *
PROFESSOR
DEPARTMENT OF SOCIOLOGY
COLLEGE OF ARTS AND SCIENCES
UNIVERSITY OF NEBRASKA-LINCOLN
LINCOLN, NE 68588

HAVENS, JENNIFER R, PHD *
ASSOCIATE PROFESSOR
DEPARTMENT OF BEHAVIORAL SCIENCE
COLLEGE OF MEDICINE
UNIVERSITY OF KENTUCKY
LEXINGTON, KY 40504

HIGHTOW-WEIDMAN, LISA B, MD, MPH
ASSOCIATE PROFESSOR
DIVISION OF INFECTIOUS DISEASES
DEPARTMENT OF MEDICINE
SCHOOL OF MEDICINE
UNIVERSITY OF NORTH CAROLINA
CHAPEL HILL, NC 27599-7030

KENDALL, CARL, PHD *
PROFESSOR
DEPARTMENT OF GLOBAL COMMUNITY HEALTH
AND BEHAVIORAL SCIENCES
SCHOOL OF MEDICINE
TULANE UNIVERSITY
NEW ORLEANS, LA 70112

KERSHAW, TRACE S, PHD
PROFESSOR
CENTER FOR INTERDISCIPLINARY RESEARCH ON AIDS
DEPARTMENT OF EPIDEMIOLOGY
SCHOOL OF PUBLIC HEALTH
YALE UNIVERSITY
NEW HAVEN, CT 06510

KURTZ, STEVEN P, PHD *
PROFESSOR AND DIRECTOR
CENTER FOR APPLIED RESEARCH ON SUBSTANCE USE
AND HEALTH DISPARITIES
DEPARTMENT OF JUSTICE AND HUMAN SERVICES
NOVA SOUTHEASTERN UNIVERSITY
CORAL GABLES, FL 33134

LEWIS, CRYSTAL FULLER, PHD *
ASSOCIATE PROFESSOR
DEPARTMENT OF PSYCHIATRY
LANGONE MEDICAL SCHOOL
NEW YORK UNIVERSITY
NEW YORK, NY 10016

MACQUEEN, KATHLEEN M, PHD *
SENIOR SCIENTIST
SOCIAL AND BEHAVIORAL HEALTH SCIENCES
FHI 360
DURHAM, NC 27701

MEEK, ERIN, DRPH *
SENIOR RESEARCH SCIENTIST
AIDS OFFICE
SAN FRANCISCO DEPARTMENT OF PUBLIC HEALTH
SAN FRANCISCO, CA 94102

MENA, LEANDRO ANTONIO, MD *
PROFESSOR AND CHAIR
POPULATION HEALTH SCIENCE
JOHN D BOWER SCHOOL OF POPULATION HEALTH
UNIVERSITY OF MISSISSIPPI MEDICAL CENTER
JACKSON, MS 39216

PAGE, KIMBERLY, PHD
PROFESSOR AND CHIEF
DIVISION OF EPIDEMIOLOGY, BIOSTATISTICS,
AND PREVENTIVE MEDICINE
DEPARTMENT OF INTERNAL MEDICINE
UNIVERSITY OF NEW MEXICO HEALTH SCIENCES CENTER
ALBUQUERQUE, NM 87131

RAMSEY, SUSAN E, PHD
ASSOCIATE PROFESSOR
DIVISION OF GENERAL INTERNAL MEDICINE
RHODE ISLAND HOSPITAL
PROVIDENCE, RI 02903

ROSENBERG, ELI SAMUEL, PHD *
ASSOCIATE PROFESSOR
DEPARTMENT OF EPIDEMIOLOGY AND BIOSTATISTICS
SCHOOL OF PUBLIC HEALTH
UNIVERSITY AT ALBANY, STATE UNIVERSITY OF NEW YORK
RENSSELAER, NY 12144

ROSSER, B R SIMON, PHD *
PROFESSOR
DIVISION OF EPIDEMIOLOGY AND COMMUNITY HEALTH
SCHOOL OF PUBLIC HEALTH
UNIVERSITY OF MINNESOTA
MINNEAPOLIS, MN 55454

SANDFORT, THEODORUS G M, PHD *
PROFESSOR
DEPARTMENT OF PSYCHIATRY
MAILMAN SCHOOL OF PUBLIC HEALTH
COLUMBIA UNIVERSITY
NEW YORK, NY 10032

SCHNEIDER, JOHN, MD, MPH
ASSOCIATE PROFESSOR
DEPARTMENT OF MEDICINE
UNIVERSITY OF CHICAGO
CHICAGO, IL 60637

SIMONI, JANE MARIE, PHD
PROFESSOR
DEPARTMENT OF PSYCHOLOGY
UNIVERSITY OF WASHINGTON
SEATTLE, WA 98195

SWEAT, MICHAEL D, PHD
PROFESSOR
DEPARTMENT OF PSYCHIATRY
AND BEHAVIORAL SCIENCES
MEDICAL UNIVERSITY OF SOUTH CAROLINA
CHARLESTON, SC 29407

TURAN, JANET M, PHD
PROFESSOR
DEPARTMENT OF HEALTH CARE ORGANIZATION
AND POLICY
SCHOOL OF PUBLIC HEALTH
UNIVERSITY OF ALABAMA AT BIRMINGHAM
BIRMINGHAM, AL 35294

MAIL REVIEWER(S)

DEL VALLE, SARA, PHD
DEPUTY GROUP LEADER
DEFENSE SYSTEMS AND ANALYSIS DIVISION
LOS ALAMOS NATIONAL LABORATORY
LOS ALAMOS, NM 87545

GOODREAU, STEVEN M., PHD
ASSOCIATE PROFESSOR
DEPARTMENT OF ANTHROPOLOGY
UNIVERSITY OF WASHINGTON
SEATTLE, WA 98105

SCIENTIFIC REVIEW OFFICER

GUERRIER, JOSE H, PHD
SCIENTIFIC REVIEW OFFICER
CENTER FOR SCIENTIFIC REVIEW
NATIONAL INSTITUTES OF HEALTH
BETHESDA, MD 20892

EXTRAMURAL SUPPORT ASSISTANT

STROTHERS, DIARA
EXTRAMURAL SUPPORT ASSISTANT
CENTER FOR SCIENTIFIC REVIEW
NATIONAL INSTITUTES OF HEALTH
BETHESDA, MD 20892

* Temporary Member. For grant applications, temporary members may participate in the entire meeting or may review only selected applications as needed.

Consultants are required to absent themselves from the room during the review of any application if their presence would constitute or appear to constitute a conflict of interest.
